# Hydrogel-based drug delivery systems for enhanced tumor therapy

**DOI:** 10.1039/d5ra08269b

**Published:** 2026-02-05

**Authors:** Shijia Tian, Shuxiang Yang, Yanfei Liu

**Affiliations:** a Key Laboratory of Cell Engineering of Guizhou Province, Affiliated Hospital of Zunyi Medical University Zunyi 563000 China l_yfei@163.com yanfei_liu@zmu.edu.cn; b Guizhou Biomanufacturing Laboratory, Affiliated Hospital of Zunyi Medical University Zunyi 563000 China; c Department of Endocrinology, The Second Affiliated Hospital of Zunyi Medical University Zunyi 563006 China

## Abstract

Cancer remains a major global health challenge, with current treatment modalities like surgery, radiotherapy, chemotherapy, targeted therapy, and immunotherapy often hampered by limitations such as systemic toxicity, drug resistance, and an immuno-suppressive tumor microenvironment (TME). Hydrogel technology emerges as a transformative platform to overcome these hurdles. The unique three-dimensional network structure of hydrogels allows for high drug loading, localized sustained release, and exceptional tunability. This review highlights the innovative applications of hydrogel systems in enhancing various antitumor modalities, including chemotherapy, immunotherapy, radiotherapy, phototherapy, gene therapy, and their combinations. By enabling spatiotemporally controlled delivery and modulating the TME, hydrogels significantly improve therapeutic efficacy while minimizing off-target effects. The purpose of this study is to describe some of the latest developments in the use of hydrogels for the treatment of cancer.

## Abstract 2

This review highlights hydrogel-based platforms for spatiotemporally controlled, localized cancer therapy, overcoming drug resistance and modulating the tumor microenvironment to enhance efficacy and reduce toxicity for multiple treatment modalities.

## Graphical abstract

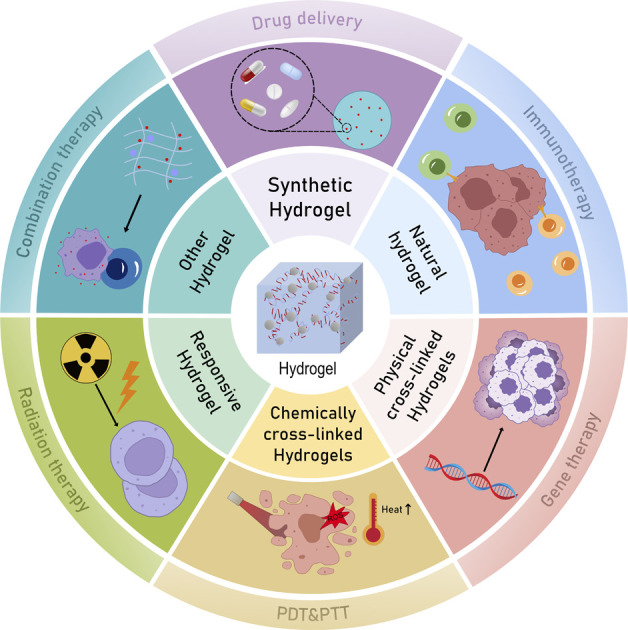

## 1 Introduction

Cancer represents a major global public health challenge, and innovation in its treatment modalities remains a cutting-edge focus of medical research.^1^ Although a comprehensive treatment strategy combining surgical resection, radiotherapy, chemotherapy, targeted therapy, and immunotherapy has been established in clinical practice, each approach possesses inherent limitations that are difficult to overcome.^2^ Surgery is highly effective for early-stage, localized tumors but struggles to eliminate micrometastases.^3^ Radiotherapy faces dose-limiting toxicities and is often ineffective against radioresistant tumors.^4^ Chemotherapy suffers from a lack of drug targeting, leading to severe systemic toxicity, significantly reducing patient quality of life through side effects such as bone marrow suppression and gastrointestinal reactions.^5^ Although targeted therapy offers improved specificity, rapid resistance development due to tumor cell gene mutations often occurs.^5^ Immunotherapy, represented by immune checkpoint inhibitors, has shown breakthroughs in some tumor types, but the overall response rate remains below 30%, and it can induce severe immune-related adverse reactions.^6^ The root cause of these challenges lies in the inability of current strategies to achieve high selectivity against tumor tissue and effective modulation of the TME. The development of drug delivery systems offers a novel approach to address this issue, and among these, hydrogel technology, with its unique tunability in physical chemistry and adaptability to biological interfaces, shows great potential for breaking through existing therapeutic bottlenecks.

Hydrogels, as the next generation of drug carriers, offer not only the advantage of overcoming the limitations of conventional delivery systems but also redefine the interaction between localized and systemic therapy. The hydrogel matrix, delivered in injectable or implantable forms to the lesion site, creates a structured drug release depot.^7–9^ Their macro-scale network structure overcomes the payload limitation bottleneck inherent in nanocarriers, while their micro-scale multi-level pores allow for fine-tuning of drug release kinetics.^10^ Importantly, modern materials engineering endows hydrogels with dynamic responsiveness, enabling them to passively adapt to TME characteristics (such as acidic pH, high reducing power, or specific enzyme expression) and actively remodel the local microenvironment by alleviating hypoxia, regulating immunosuppression, or restructuring the extracellular matrix (ECM).^11–13^ This bidirectional interaction characteristic allows the hydrogel system to serve as a “toxicity reduction and efficacy enhancement” carrier for conventional chemotherapeutic drugs and also as an ideal delivery platform for immunomodulators, gene editing tools, and other novel therapeutic molecules, providing the material basis for developing breakthrough combination therapies.

In short, hydrogel systems hold significant potential for cancer therapy. This review examines various hydrogel-based platforms capable of efficient tumor targeting and highlights their key therapeutic advantages. The aim is to provide insights and inspiration for developing more precise, effective, and personalized cancer treatments.

## 2 Methods

The literature search was conducted using the keywords “hydrogel,” “drug delivery,” “tumor therapy,” “antitumor,” and “sustained release,” combined with subject heading expansion. Databases including PubMed and Elsevier ScienceDirect were searched for relevant literature published between 2021 and 2025. Original research articles or reviews focusing on hydrogel-based drug delivery systems in tumor therapy, as well as studies investigating drug-controlled release mechanisms and *in vitro*/*in vivo* efficacy validation, were included. Duplicate publications or studies with incomplete data were excluded.

## 3 Classification characteristics of hydrogels

**Fig. 1:**
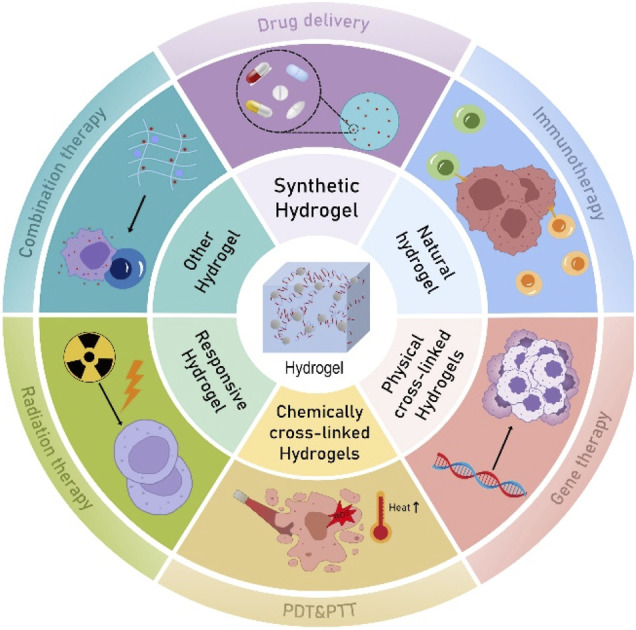
Schematic of hydrogel classifications for oncology therapeutic applications.

Hydrogels are a class of polymeric materials characterized by a porous, water-swollen architecture, possessing unique hydrophilicity and swelling properties that confer widespread application potential in fields such as biomedical engineering, flexible electronics, and environmental engineering.^14^ Based on their chemical composition, crosslinking methods, and response characteristics, hydrogels can be systematically classified (Fig. 1). Regarding chemical composition, hydrogels are primarily divided into two categories: natural polymer hydrogels and synthetic polymer hydrogels. Natural polymer hydrogels typically originate from biological sources or biopolymer fermentation processes, including collagen,^15,16^ hyaluronic acid (HA),^17^ fibrinogen,^18^ chitosan,^19,20^ sodium alginate (SA),^21^*etc.* These materials offer unparalleled advantages in biomedical applications such as tissue engineering scaffolds, wound dressings, and drug delivery systems due to their inherent biocompatibility, low immunogenicity, and biodegradability.^22^ For instance, collagen hydrogels can mimic the structure and function of the native ECM, promoting cell adhesion and proliferation;^23^ HA hydrogels excel in applications like intra-articular injections and ophthalmic uses due to their good water-retention capacity and lubricating properties.^24^ In contrast, synthetic polymer hydrogels, such as polyacrylamide (PAAm), polyethylene glycol (PEG), polyvinyl alcohol (PVA), *etc.*, enable precise performance modulation through molecular design. Their mechanical strength, degradation rate, and functional groups can be tailored *via* polymerization reactions, endowing them with unique value in emerging fields like flexible sensors, artificial muscles, and biomimetic materials.

Based on crosslinking mechanisms, hydrogels are categorized into physically crosslinked and chemically crosslinked hydrogels. Physically crosslinked hydrogels form dynamic network structures through non-covalent interactions, such as hydrogen bonding, hydrophobic interactions, ionic crosslinking, crystalline domain crosslinking, and host–guest interactions. The key feature of these hydrogels is their reversibility; under external stimuli (*e.g.*, temperature, pH, ionic strength changes), they can undergo reversible gel–sol transitions.^25^ This property makes them suitable for applications like self-healing materials and smart drug delivery systems. For instance, Pluronic® triblock copolymer-based thermosensitive hydrogels undergo sol–gel transitions near body temperature, suitable for localized drug delivery;^26,27^ SA hydrogels crosslinked by Ca^2+^ ions are widely employed for cell encapsulation due to their mild gelation conditions. Chemically crosslinked hydrogels, on the other hand, form permanent network structures *via* covalent bonds, using methods like free radical polymerization, click chemistry, enzyme-catalyzed crosslinking, *etc.* These hydrogels generally exhibit higher mechanical strength and structural stability. For example, the elastic modulus of PAAm hydrogels can reach MPa levels, making them suitable for biomedical applications requiring long-term mechanical support. Notably, the recently developed double-network hydrogels have enabled unprecedented mechanical robustness through combined physical and chemical crosslinking strategies, significantly expanding their application in load-bearing tissue replacement and related fields.^28^

Based on their response behavior to external stimuli, hydrogels can also be further subdivided into various types of smart, stimulus-responsive materials.^29,30^ Thermoresponsive hydrogels like poly(*N*-isopropylacrylamide) (PNIPAM) exhibit significant volume phase transitions near their lower critical solution temperature (LCST), a property exploited for designing temperature-switch drug delivery systems. pH-responsive hydrogels, such as polyacrylic acid derivatives, show differential swelling under the diverse pH conditions in the gastrointestinal tract, enabling targeted drug delivery.^31^ Photothermal-responsive hydrogels incorporate photosensitive groups like azobenzene or spiropyran, allowing remotely controlled gel behavior.^32^ Enzyme-responsive hydrogels can specifically respond to proteases like matrix metalloproteinases (MMPs), offering unique advantages in TME-responsive drug delivery.^33^ These smart hydrogels can perceive and respond to complex physiological environmental changes, providing powerful tools for precision medicine and controlled release. With the advancement of supramolecular chemistry and dynamic covalent chemistry, smart hydrogel systems possessing multi-responsive characteristics, adaptability, and self-healing functions are continuously emerging, further driving the depth and breadth of research in this field.

## 4 Sustained release mechanisms of hydrogels in tumor therapy

Traditional chemotherapy drugs often face challenges such as high systemic toxicity, poor tumor targeting, drug burst release, and difficulty overcoming the TME barrier. Hydrogel, as drug delivery systems for tumor therapy, demonstrate significant potential that surpasses conventional delivery methods due to their physicochemical properties and highly designable biological functionalities.^34^

As an efficient drug delivery system, the sustained-release performance of hydrogels relies on the synergistic effects of various physicochemical mechanisms to achieve controlled drug release from the carrier to the target tissue. Drug release from hydrogels is not a simple escape process but rather a complex kinetic process regulated by the properties of the material itself, drug characteristics, and the external environment. Currently, the release mechanisms can be primarily categorized into passive diffusion-controlled release and active stimulus-responsive release. The former depends on the drug molecule's own diffusion and carrier degradation, while the latter can respond to specific stimuli from the TME or external sources, enabling more precise and intelligent on-demand drug delivery.^35,36^ A deep understanding of these mechanisms is crucial for designing efficient and safe tumor treatment strategies.

### 4.1 Passive diffusion-controlled release

Passive diffusion-controlled release is the most fundamental and widely used drug release mechanism. Its driving force mainly comes from the chemical potential gradient. The release rate is primarily governed by the physical structure of the hydrogel network and mainly includes three modes: diffusion-controlled release, degradation-controlled release, and swelling-controlled release.^37^

#### 4.1.1 Diffusion-controlled released

This mechanism applies when drug molecules are physically encapsulated within the hydrogel network. The drug dissolves in the hydrophilic matrix of the hydrogel and diffuses into the external environment through microscopic pores or aqueous channels between polymer chains. The release kinetics generally follow Fick's law of diffusion, where the release rate is proportional to the square root of time, representing a non-constant release profile. The drug release rate from hydrogels is governed by several key factors. Firstly, the crosslinking density dictates the network's mesh size, where a highly crosslinked structure reduces pore dimensions and slows diffusion.^38^ Secondly, the physicochemical characteristics of the drug molecule are critical; small molecules diffuse rapidly,^39–41^ whereas larger entities like monoclonal antibodies^42,43^ or cytokines such as IL-2 and interferon^44^ experience significant steric hindrance. Thirdly, the swelling degree of the hydrogel influences release, as a more swollen state expands the polymer network, increases pore size, and creates more open diffusion pathways, thereby accelerating release,^45,46^ Additionally, drug–polymer interactions, such as electrostatic forces and hydrogen bonding, also influence the diffusion rate.

A major issue with this mechanism is the initial burst release effect, which occurs due to the direct contact of surface-layer drugs with the release medium and the rapid hydration of network pores. This may cause acute toxic side effects and shorten the effective treatment duration, as well as make it difficult to achieve long-term release, thereby affecting the stability and predictability of the therapeutic outcome. Strategies to mitigate burst release include multi-layer coating, increasing crosslinking density, or optimizing drug loading methods.^47–49^

#### 4.1.2 Degradation-controlled release

Pore diffusion in drug sustained-release is often accompanied by degradation-controlled release. In the initial phase of drug release, diffusion often dominates. As the gel degrades, the release mechanism transitions to being degradation-controlled. At this stage, drug release is no longer a simple diffusion process but the result of two consecutive and interrelated processes: the biodegradation of the hydrogel carrier and the subsequent release of the entrapped drug *via* diffusion or matrix erosion.

Degradation modes mainly include hydrolysis and enzymatic degradation. Hydrolytic degradation involves the chemical cleavage of functional groups like ester, amide, or anhydride bonds in the aqueous environment of bodily fluids. Its rate depends on the bond type, surrounding pH, and temperature. Poly(lactic-*co*-glycolic acid) (PLGA)-based hydrogels are typical examples. The degradation rate of ester bonds in PLGA hydrogels is related to environmental pH,^37^ the lactic acid to glycolic acid ratio,^50^ and its intrinsic viscosity.^51^ Therefore, modulating these parameters can control the sustained-release kinetics to match the treatment cycles of different diseases. Enzymatic degradation-controlled release is discussed later.

A significant advantage of this mechanism is that its release kinetics can potentially achieve near-zero-order release kinetics, and thus maintain a relatively constant drug release rate over an extended period. However, since this method typically requires incorporating degradable functional groups into the polymer chains or using them as crosslinkers, the biosafety of the degradation products needs comprehensive evaluation, including their metabolic pathways, cytotoxicity, and potential for inflammatory responses, to ensure clinical application safety.^52^

#### 4.1.3 Swelling-controlled release

Swelling-controlled release is a drug release mechanism that relies on the physical state transition of the hydrogel. The process begins with the hydrogel in a dry/shrunken state, such as a glassy or semi-crystalline state. When the hydrogel is implanted or injected into the physiological environment, water molecules hydrate the hydrophilic groups on the polymer chains. This process disrupts secondary bonds (*e.g.*, hydrogen bonds, van der Waals forces) between chains, enhancing chain segment mobility, and gradually relaxes and expands the network structure. As the hydrogel swells, its mesh pore size increases significantly, and the internal osmotic pressure changes, creating diffusion pathways for the entrapped drug molecules. The drug is then released into the external environment following the concentration gradient. Common swelling materials include PLGA-based hydrogels, gelatin, alginate/chitosan hydrogels, *etc.*^53–55^

### 4.2 Active stimulus-responsive release

To address the complexity of tumor treatment and improve targeting and controllability of drug release, “smart” hydrogels have emerged. They can perceive specific stimulus signals from the TME or external applications and undergo significant physical form or chemical structure changes, thereby actively triggering on-demand, localized, and quantitative drug release. This enhances treatment precision, efficiency, and reduces systemic side effects.^56^

#### 4.2.1 Physical stimulus-responsive release

This type of release is triggered by externally applied physical energy, offering non-invasive or minimally invasive remote control means with good spatiotemporal controllability.

Temperature-responsive hydrogels are among the most extensively studied physical response systems. They are typically composed of thermosensitive polymers, with pNIPAM and its derivatives being classic examples.^57^ Others include PEO–PPO–PEO triblock copolymers like Pluronic F127,^58^ chitosan,^59^ and gelatin.^60^ When the environmental temperature is below the LCST, the polymers form hydrogen bonds with water molecules and remain in a swollen state. When heat is applied *via* external sources or combined with photothermal therapy (PTT), hydrogen bonds are disrupted. The polymer chains undergo a sharp hydrophilic–hydrophobic phase transition, dehydrate, and transition to a collapsed state. This volume phase transition can effectively squeeze out and release a large amount of the encapsulated drug, achieving switch-like control.^61^

Light-responsive release strategies utilize light as a trigger. It is primarily achieved through two pathways: first, incorporating photothermal conversion nanomaterials such as gold nanorods,^62^ black phosphorus nanosheets^63^ and carbon nanotubes.^64^ Under near-infrared (NIR) irradiation, these nanomaterials generate heat, subsequently inducing a phase transition in thermosensitive hydrogels to release drugs. Second, modifying polymer chains with photosensitive chemical groups such as *o*-nitrobenzyl esters, pyrenyl and azobenzene.^65^ These groups undergo photolysis or isomerization upon photon absorption, leading to chemical bond cleavage or molecular conformation changes, thereby degrading the hydrogel or altering its hydrophilicity/hydrophobicity to release drugs. This method offers remarkably high precision, enabling spatial resolution at the millimeter scale.

Ultrasound-responsive release utilizes the energy of ultrasonic mechanical waves. The cavitation, thermal, and mechanical effects of ultrasound can physically disrupt the physical crosslinks or dense structure of hydrogels, enhance the mobility of polymer chains and drug diffusion capacity, thus achieving pulsed or enhanced drug release.^66^ The strong penetration depth of ultrasound makes it suitable for treating deep-seated tumors. Magnetic Field-Responsive release typically requires embedding superparamagnetic nanoparticles within the hydrogel, such as Fe_3_O_4_ (ref. 67) and ferromagnetic vortex-domain iron oxide.^68^ When an external alternating magnetic field is applied, the magnetic particles generate heat through hysteresis loss or Néel relaxation,^69^ thus triggering drug release from the hydrogel.

Physical stimulus-responsive release offers high precision and low side effects, but its clinical application often relies on external equipment, increasing treatment complexity and cost. Simultaneously, tissue penetration depth, distribution uniformity of the energy, and long-term safety are key issues requiring continuous optimization and consideration.

#### 4.2.2 Chemical stimulus-responsive release

This release mechanism utilizes the inherent chemical differences between the TME and normal tissues as intrinsic trigger signals. It is a primary strategy for achieving autonomous targeting and smart release without external intervention, offering high specificity and biocompatibility.

pH-responsive release is based on the unique acidic microenvironment of tumor tissues. Due to the Warburg effect, tumor cells favor high-rate glycolysis, producing large amounts of lactic acid,^70^ leading to a significantly lower extracellular pH compared to normal tissue. Furthermore, intracellular endosomal and lysosomal compartments are even more acidic (pH 4.5–5.5).^71^ Targeting this characteristic, one approach uses pH-sensitive chemical bonds, such as hydrazone bonds,^72^ ketal bonds,^73^ boronic ester bonds^74^ and imine bonds,^75^ as linkers to covalently conjugate drug molecules to the polymer backbone. These bonds are stable at neutral pH but rapidly hydrolyze and break in the acidic environment, releasing the free drug. The other approach uses inherently pH-responsive polyelectrolyte polymers, such as anionic hydrogels like poly(acrylic acid)^76^ and SA,^77^ as well as cationic hydrogels like chitosan.^78^ The ionizable groups on their chains protonate under acidic conditions, causing drastic changes in polymer chain conformation or solubility, thereby accelerating drug diffusion release.

Redox-responsive release cleverly exploits the difference in redox potential between the tumor cell cytosol and extracellular fluid. To maintain rapid proliferation and combat oxidative stress, the intracellular concentration of GSH in tumor cells can be 100 to 1000 times higher than the extracellular concentration (about 2–10 mM intracellular *vs.* 2–20 µM extracellular).^79^ This steep gradient provides a robust trigger for intracellular drug release. The disulfide bond (–S–S–) is a typical reduction-sensitive linker, which undergoes rapid cleavage to thiols in the presence of high GSH concentrations, leading to hydrogel degradation or drug liberation.^80^ Selenocystine (–Se–Se–) bonds, analogous to disulfides but with higher sensitivity to mild reductants, have also been explored for redox-triggered release.^81^ Concurrently, the high ROS environment generated by the Warburg effect in tumor tissue can also be harnessed as an exploitable trigger for extracellular or tumor-selective drug release.^82^ This oxidizing state, featuring elevated levels of species such as hydrogen peroxide (H_2_O_2_) and hydroxyl radicals (–OH), drives specific chemical transformations in engineered hydrogels. Thioether bonds (–C–S–C–) are a primary target, undergoing oxidation to hydrophilic sulfoxides or sulfones that induce hydrogel dissolution.^83^ Other responsive motifs include boronic ester bonds, which are cleaved specifically by H_2_O_2_,^84,85^ and thioketal bonds (–S–C–S–),^86^ which are selectively broken down by –OH. Beyond these static chemical triggers, advanced self-amplifying systems have been developed where initially generated ROS further activates the bulk release from the ROS-sensitive matrix, creating a potent feedback loop for enhanced therapy.^87,88^

Enzyme-responsive release utilizes specific enzymes that are often overexpressed or abnormally active in the TME as trigger signals. These enzymes include MMPs involved in ECM remodeling,^89^ cathepsins associated with cell proliferation,^90^ and hyaluronidase,^91^ among others. The design strategy involves integrating sequences that can be specifically recognized and cleaved by these enzymes (*e.g.*, the substrate peptide GPLGIAGQ for MMPs^92^) as crosslinkers into the hydrogel network or as linking units between the drug and the carrier. In the enzyme-rich TME, enzymatic hydrolysis of the hydrogel degrades its network or cleaves its linkers, enabling the specific activation and release of the drug.

Chemically responsive drug release can autonomously target diseased areas and differentiate them from healthy tissue without external devices. However, its efficacy highly depends on the intensity and homogeneity of the stimulus signals in the TME. The TME itself is highly heterogeneous; significant variations in pH, GSH concentration, or enzyme activity may exist between different patients, different tumor types, or even different regions of the same tumor,^93,94^ which might affect the drug release kinetics and treatment uniformity. Therefore, combining multiple chemical stimuli responses or combining chemical and physical responses is common to overcome the limitations of single mechanisms and achieve more robust targeted release.

## 5 Hydrogel-based drug delivery systems for antitumor therapies

### 5.1 Chemotherapy

Hydrogel delivery systems demonstrate significant advantages in improving the performance of antitumor drugs. Many first-line antitumor drugs currently used clinically suffer from issues such as poor water solubility, low cellular uptake efficiency, insufficient targeting, and significant toxicity,^95–97^ which limit their therapeutic effects. Using hydrogels as drug carriers can effectively address these problems. Their hydrophilic network encapsulates hydrophobic drugs, improving both solubility and stability in the physiological microenvironment. Through localized, sustained release, hydrogels maintain high drug concentrations at the tumor site. This concentration gradient facilitates passive diffusion of the drug into tumor cells, thereby overcoming the low uptake rates typically associated with systemic administration. Moreover, the design flexibility of hydrogels allows the incorporation of targeting moieties or the engineering of stimuli-responsive release mechanisms triggered by the TME, thereby improving targeting precision in both space and time.^98^ Guo *et al.* developed a cyclen-modified self-assembled peptide hydrogel (FFFK-cyclen) for loading chlorambucil (CRB). Cyclen incorporation notably enhances the water solubility and degradation resistance of CRB, overcoming its susceptibility to breakdown by body fluid. Under the acidic TME, cyclen readily becomes protonated, conferring a positive charge to the hydrogel. This enhances its affinity for negatively charged cell membranes and promotes cellular uptake. Compared to free CRB, the CRB-loaded hydrogel system exhibited stronger tumor-killing effects, effectively inducing tumor cell DNA damage while maintaining good biocompatibility.^99^ Similarly, the hydrophobic fisetin was encapsulated within the peptide hydrogel RADA16-I, forming an *in situ* drug-loading system that enhanced antitumor efficacy while enabling controlled release. By adjusting the peptide concentration, the drug release rate could be precisely controlled.^100^ Concurrently, the curcumin-loaded RADA16-I hydrogel system demonstrated comparable advantages through significant tumor growth inhibition (TGI) and markedly reduced toxicity to normal cells.^101^ These studies fully demonstrate that hydrogel carriers can effectively enhance drug stability, achieve controlled release, and enable targeted delivery, thereby significantly reducing the damage of antitumor drugs to healthy tissues. Li *et al.* prepared a heparin-pluronic nanogel (HP) by conjugating low molecular weight heparin with carboxylated Pluronic P407, which was used to load doxorubicin (DOX), forming a drug-loaded system (DOX@LR-HP).^102^ In this study, DOX was first pre-adsorbed onto magnesium silicate nanosheets (LAPONITE^®^ RDS, LR) *via* ion exchange. Subsequently, this complex was combined with the HP polymer to construct a thermosensitive hydrogel with sustained-release functionality. Results showed that DOX@LR-HP exhibited a more gradual and controllable release profile over 21 days, whereas the DOX@HP and DOX@P-407 groups, lacking the LR carrier, displayed varying degrees of burst release. In cellular experiments, DOX@LR-HP demonstrated the strongest proliferation inhibition and cytotoxicity against S180 sarcoma cells. Furthermore, in S180 xenograft tumor model, DOX@LR-HP exhibited optimal antitumor efficacy by inhibiting CD31-associated angiogenesis and cancer cell proliferation.

**Fig. 2:**
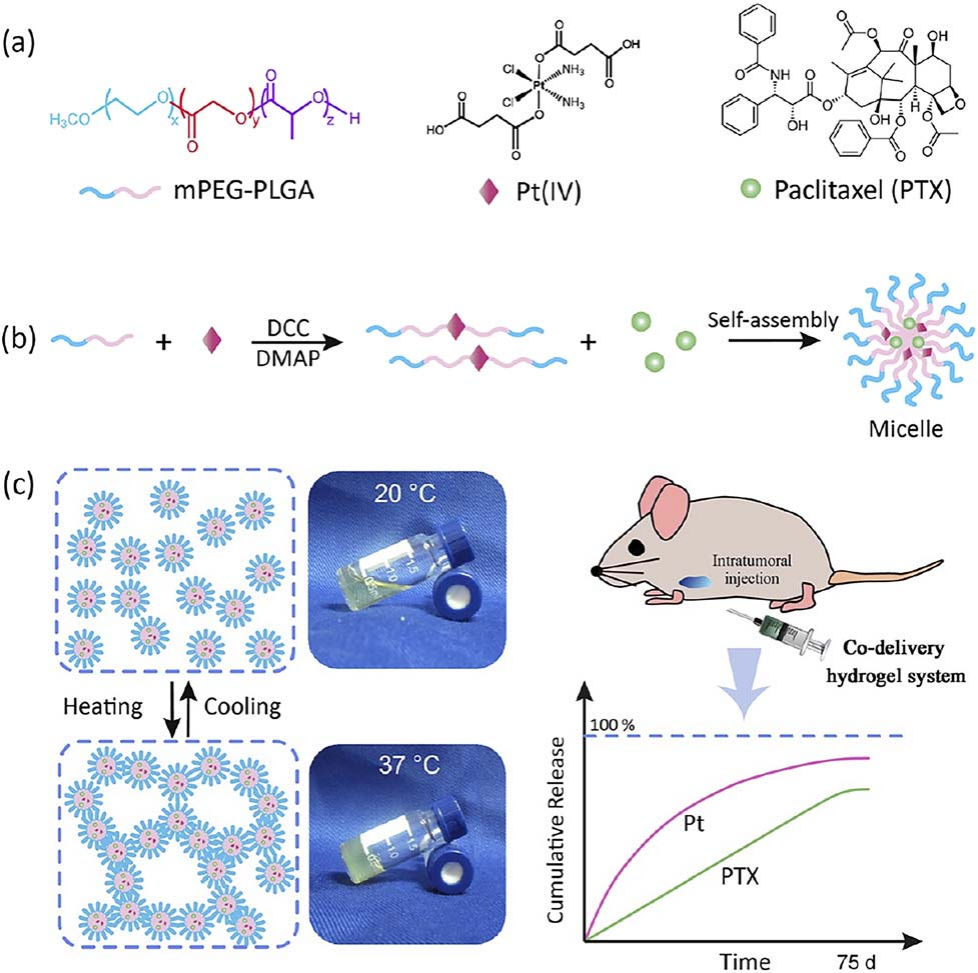
Design of hydrogel formulation for the combination delivery of cisplatin and PTX. (a) Molecular structures of the mPEG–PLGA diblock copolymer, Pt(iv) prodrug, and PTX. (b and c) A polymer–Pt(iv) conjugate was synthesized by covalently linking mPEG–PLGA diblock copolymers onto a Pt(iv) prodrug. The resultant conjugates could self-assemble into micelles in water and further exhibited a sol–gel transition upon heating and therefore could serve as the carrier for PTX codelivery, reproduced from ref. 109 with permission from American Chemical Society (*ACS Appl. Mater. Interfaces*, 2017), copyright 2017.

Single-drug treatment regimens often face the challenge of developing drug resistance in cancer therapy. Combination therapy using two or more antitumor drugs has been proven to mitigate drug resistance, enhance therapeutic effects, reduce toxicity associated with single-drug dosing, and expand the therapeutic window of drugs.^103–105^ Hydrogels can also achieve the synchronized delivery of multiple drugs and allow for improved spatiotemporal control over the distribution of each drug, thereby providing novel cancer treatment strategies with superior efficacy and controllable toxicity for clinical use.^106^ Many studies have fully validated the excellent performance of hydrogels in combination drug delivery. Karavasili *et al.* developed a DOX and curcumin co-loaded self-assembled peptide hydrogel system that showed significant advantages in head and neck tumor therapy.^107^ Compared to the free drug combination, the co-loaded system demonstrated superior inhibitory effects on tumor cell proliferation and enhanced apoptosis induction. Specifically, tumor cell apoptosis was induced even at drug concentrations substantially below the individual IC50 values. An *in situ* thermosensitive Pluronic F127-based hydrogel was designed for the co-delivery of DOX and docetaxel (DOC) through the simultaneous encapsulation of a DOX–HA nanocomplex and DOC-loaded pluronic mixed micelles within its matrix.^108^ After intratumoral administration of this formulation in a tumor-bearing mouse model, significant tumor suppression was observed. The mixed micelle-hydrogel group achieved a tumor inhibition rate of 92.4%, significantly higher than those of free DOX (75.6%), DOC solution (50.6%), and DOC-micelle groups (49.5%). Pharmacokinetic results confirmed that this formulation prolonged the retention time of both DOX and DOC at the tumor site while reducing systemic toxicity. Biodistribution experiments also revealed that the accumulation of DOX from the intratumoral hydrogel was 14.9 times that of the free drug. In another study, Shen *et al.* developed an injectable thermosensitive prodrug hydrogel system for the synergistic delivery of cisplatin (cDDP) and paclitaxel (PTX) to treat ovarian cancer (Fig. 2).^109^ The researchers first conjugated mPEG–PLGA with a platinum(iv) prodrug *via* covalent bonds, forming a bimolecular mPEG–PLGA–Pt(iv) conjugate. Subsequently, hydrophobic PTX was loaded into this amphiphilic conjugate, forming core–shell structured micelles. Experiments demonstrated that the two drugs could achieve sustained release for up to 2.5 months. Both *in vitro* and *in vivo* studies confirmed that the dual-drug-loaded conjugate hydrogel system exhibited synergistic and enhanced antitumor activity. Beyond dual–drug combinations, hydrogels have also been employed for more complex regimens. For instance, a thermosensitive PLGA–PEG–PLGA hydrogel was used to co-deliver the three chemotherapeutics DOX, cDDP, and methotrexate against osteosarcoma.^110^ Peritumoral administration of this triple-drug-loaded hydrogel in a murine model resulted in sustained tumor inhibition over 16 days, accompanied by enhanced apoptosis, without inducing significant systemic toxicity. This case illustrates the capability of hydrogels to facilitate complex multidrug regimens while preserving biocompatibility.

Local chemotherapy is one of the primary treatment modalities for advanced cancer patients, and faces a major therapeutic bottleneck due to drug penetration barriers. A tubustecan hydrogel was developed by covalently conjugating the tissue-penetrating cyclic peptide iRGD to two camptothecin (CPT) units, creating a prodrug amphiphile that self-assembles into hollow tubular nanostructures. These nanotubes form an injectable hydrogel depot *in situ*, enabling the simultaneous loading of DOX and curcumin for combination therapy.^111^ The conjugated iRGD peptide facilitates tumor targeting and tissue penetration^112^ through a two-step mechanism: it initially binds to αv integrins on tumor vasculature, and following proteolytic cleavage, the resulting RGDR sequence further promotes deep tumor penetration and cellular internalization by binding to neuropilin-1. *In vitro* and *in vivo* studies confirmed that this co-drug hydrogel could significantly enhance the permeability of chemotherapeutic drugs into tumor tissue, enabling sustained drug release, thereby effectively inhibiting tumor metastasis and recurrence while alleviating off-target toxicity.

### 5.2 Immunotherapy

Immunotherapy represents a revolutionary treatment strategy that activates the patient's own immune system to recognize and kill tumor cells. Compared to traditional surgery, radiotherapy, and chemotherapy, it has developed into an essential modality in antitumor therapy, demonstrating significant efficacy and unique advantages in clinical practice.^113,114^ Hydrogels as carriers for immunotherapy are receiving increasing attention. Encapsulating or loading immunomodulators or immune cells into hydrogels enables controlled and sustained release as well as localized enrichment at the tumor site. This approach effectively overcomes the bottlenecks associated with systemic administration, such as off-target toxicity, short half-life, and an immunosuppressive TME. This hydrogel-based delivery strategy can significantly enhance immune activation, remodel the tumor immune microenvironment, and improve the intensity and durability of antitumor immune responses. Moreover, it maximizes the reduction of immune-related adverse reactions, offering a highly promising platform for enhancing efficacy and reducing toxicity in immunotherapy.

**Fig. 3:**
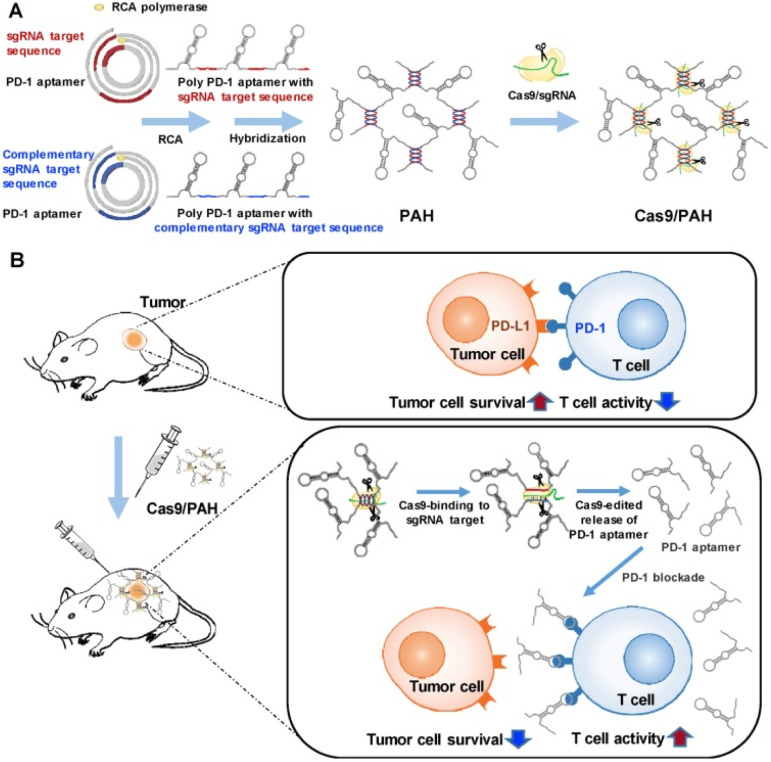
Illustration of the Cas9/sgRNA-edited immune check point-blocking DNA polyaptamer hydrogel and its action mechanism. (A) To construct a Cas9/sgRNA-edited immune checkpoint-blocking DNA polyaptamer hydrogel, two types of RCA templates were designed. Both contained the PD-1 DNA aptamer sequence, but they differed in that one contained the sgRNA target sequence for Cas9/sgRNA-specific cleavage, while the other contained the complementary sgRNA target sequence. The two RCA products were crosslinked by sequence-specific hybridization between the sgRNA target and complementary sequences. (B) In the TME, the precise excision of the PD-1 aptamer by Cas9/sgRNA can block the interaction between immune cell-surface PD-1 and tumor cell-surface PD-L1, enhancing immune cell activation for immunotherapy, reproduced from ref. 120 with permission from Elsevier (*Biomaterials*, 2019), copyright 2019.

Among various immunotherapy approaches, immune checkpoint inhibitors are the most widely used clinically. These drugs block the inhibitory effect of immune checkpoints on T cells within tumor cells or the microenvironment, reactivating the immune system's recognition and killing ability against tumors.^115,116^ Xu *et al.* developed an injectable SA-based hydrogel system to enhance T cell infiltration and block PD-L1 activity, thereby achieving efficient cancer immunotherapy.^117^ This SA-based hydrogel contained particles of linagliptin and BMS-202 loaded within its micropores. After injection into the tumor site and gelation, linagliptin effectively inhibited the degradation of the chemokine CXCL10, thereby maintaining the persistent chemotactic effect of exogenously added CXCL10 and significantly promoting T cell infiltration into the tumor area. Simultaneously, BMS-202 irreversibly inhibited the activity of PD-L1 on tumor cell surfaces, blocking the PD-L1-mediated immune escape mechanism. Results indicated that this system, combined with CXCL10, produced potent immunotherapeutic effects against both primary and distal tumors and significantly inhibited the formation of lung metastases. However, as monotherapy, immune checkpoint inhibitors exhibit low response rates.^118,119^ Addressing this issue, Lee *et al.* designed a programmable release DNA hydrogel system cleavable by Cas9/sgRNA for delivering Programmed Cell Death Protein 1 (PD-1) DNA aptamer. This hydrogel, capable of programmable release of immune checkpoint blockers DNA aptamers, could be precisely cut by Cas9/sgRNA (Fig. 3).^120^ Compared to free PD-1, the system improved the aptamer's stability, reduced its immunogenicity, and significantly increased its retention time in the TME. Consequently, it significantly improved antitumor efficacy and survival. Short rod-like nano-hydroxyapatite (nHA) can be used to upregulate PD-L1 (CD274) mRNA expression in melanoma cells. The HA hydrogel, loaded with both nHA and a PD-1/PD-L1 inhibitor, effectively suppressed tumor occurrence and growth and reversed the abnormally elevated levels of lactate dehydrogenase, aspartate aminotransferase, and alanine aminotransferase. Ultimately, it prolonged the survival of the tumor-bearing mice.^121^ CTLA-4, another important immune checkpoint molecule, primarily expressed on CD4^+^ helper T cells and cytotoxic T cells.^122^ A thermosensitive P407 hydrogel loaded with anti-CTLA-4 antibody demonstrated better biocompatibility at the tumor site and TGI in both *in vivo* and *in vitro* compared to conventional adjuvants.^123^ Certain peptide molecules also possess the ability to block PD-1/PD-L1 interaction. Compared to monoclonal antibodies, these peptides offer advantages such as smaller molecular weight, stronger tumor tissue penetration, lower immunogenicity, and lower production costs. Li *et al.* designed a d-peptide OPBP-1 capable of blocking PD-1/PD-L1 interaction and encapsulated it into *N*,*N*,*N*-trimethyl chitosan hydrogel, improving the oral bioavailability and exhibiting good tumor-suppressive effects of OPBP-1.^124^

Beyond immune checkpoint blockade therapy, hydrogels also provide an ideal delivery and functional maintenance platform for other immunotherapy modalities, including adoptive cell therapy, cytokine therapy, and cancer vaccines. The multifunctional peptide hydrogel FEFKLDV is capable of mimicking the lymph organ environment. By integrating mechanotransduction and chemical signal regulation, it accelerated CAR-T cell proliferation. CAR-T cells delivered *via* hydrogel exhibited longer retention times, stronger tumor-suppressive effects, and higher effector T cell infiltration rates.^125^ A gelatin hydrogel was used to simultaneously encapsulate IL-12/IL-15 oncolytic adenoviruses and CIK cells.^126^ This platform enabled sustained release of therapeutic components at the tumor site, thus avoiding the need for multiple injections and enhancing antitumor efficacy through synergistic action. Moreover, a PEG-*b*-poly(l-alanine) hydrogel was designed to deliver tumor cell lysate as a vaccine and granulocyte-macrophage colony-stimulating factor as an adjuvant, along with immune checkpoint inhibitors.^127^ By enhancing antigen-presenting cells' ability to uptake antigens, this system effectively improved the immunotherapy effect against melanoma and 4T-1 tumor.

### 5.3 Chemo-immunotherapy

Hydrogels can also be used to simultaneously deliver immunomodulators and antitumor drugs. By modulating the activity of the immune system and the sensitivity of tumors to drugs, these systems can enhance therapeutic effects while reducing adverse reactions in patients. Alginate hydrogels were utilized in to simultaneously deliver the antitumor drug celecoxib and the PD-1 antibody. Results confirmed that this dual delivery strategy generated sustained and potent antitumor effects both *in vitro* and *in vivo*. The system not only enhanced the immunological activity of effector T cells but also effectively alleviated immunosuppressive states, significantly improved the tumor inflammatory microenvironment, and suppressed pathological angiogenesis.^128^ Elastin-like polypeptides hydrogels were employed in to simultaneously deliver gemcitabine and anti-PD-L1 antibodies. This system increased tumor-infiltrating CD8^+^ T cells by 3.0-fold and cleared 60% of regulatory T cells, eliciting a robust anti-tumor immune response.^129^

**Fig. 4:**
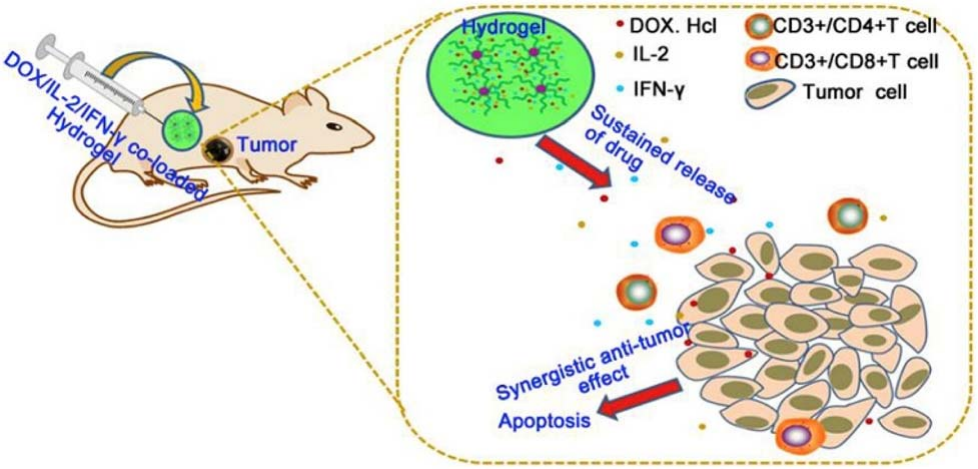
Schematic illustration for the mechanism of DOX/IL-2/IFN-γ co-loaded hydrogel of PELG7–PEG45–PELG7 on melanoma local treatment in nude mice: the released drugs from the hydrogel could induce the cell apoptosis as well as promote the proliferation of CD3/CD4 T-lymphocytes and CD3/CD8 T-lymphocytes to cause enhanced anti-tumor efficacy against melanoma xenograft in mice model, reproduced from ref. 44 with permission from KeAi (*Bioact. Mater.*, 2018), copyright 2018.

**Fig. 5:**
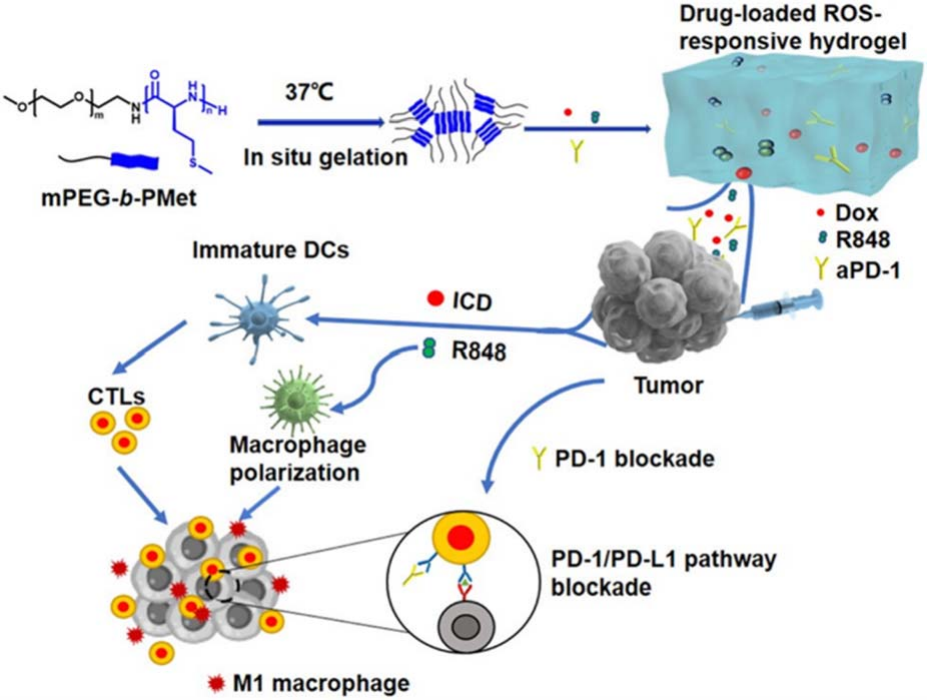
Schematic figure of a drug-loaded hydrogel *in situ* delivery system for chemoimmunotherapy on tumors, reproduced from ref. 130 with permission from Royal Society of Chemistry (*Biomater. Sci.*, 2024), copyright 2024.

In another study, Lv *et al.* designed a thermoresponsive hydrogel based on poly(γ-ethyl-l-glutamate)–poly(ethylene glycol)–poly(γ-ethyl-l-glutamate) (PELG–PEG–PELG) for the co-loading of DOX and the cytokines IL-2 and IFN-γ, exploring its potential application in combined chemo-immunotherapy for melanoma (Fig. 4).^44^ Results showed that this multi-drug co-loaded hydrogel exhibited stronger cytotoxicity against B16F10 cells *in vitro* compared to the single DOX treatment group or the group containing only IL-2/IFN-γ. Further apoptosis assays indicated that this composite gel successfully increased the apoptosis rate of tumor cells and regulated the expression of apoptosis-related genes, including downregulating the anti-apoptotic factor Bcl-2 and upregulating the protein level of Caspase-3. Additionally, compared to hydrogels loaded only with DOX or only with cytokines, the co-loaded system more effectively induced cell cycle arrest at the G2/S phase. In *in vivo* tumor inhibition experiments, the DOX/IL-2/IFN-γ co-loaded hydrogel group achieved a TGI rate of 91.51%, significantly higher than the DOX solution group (56.08%), the DOX-loaded hydrogel group (57.40%), and the cytokine-only hydrogel group (28.22%), without causing significant systemic toxicity. Mechanistic studies suggested that this synergistic antitumor effect might be closely related to the increased infiltration of CD3^+^/CD4^+^ and CD3^+^/CD8^+^ T lymphocytes within the TME. Meanwhile, Li *et al.* developed a reactive oxygen species (ROS)-responsive thermosensitive hydrogel based on a PEG–polypeptide copolymer, co-loaded with the DOX, the immunomodulator resiquimod (R848), and the anti-PD-1 for local chemo-immunotherapy (Fig. 5).^130^ This hydrogel demonstrated controllable degradation and sustained drug release *in vitro* depending on ROS concentration. Intratumoral injection in a B16F10 melanoma mouse model significantly inhibited tumor growth, prolonged mouse survival, effectively activated antitumor immune responses, and did not cause significant systemic toxicity. In a postoperative model, this composite hydrogel system showed excellent efficacy in preventing tumor recurrence and could induce long-term immune memory.

### 5.4 Radiotherapy and phototherapy

Hydrogels also serve as versatile platforms for enhancing energy-based tumor therapies, primarily radiotherapy and phototherapy. While radiotherapy employing ionizing radiation remains a cornerstone for localized tumors, phototherapy—including PTT and photodynamic therapy (PDT)—has emerged as a promising minimally invasive strategy. PTT kills tumor cells by converting light energy into heat using photothermal agents, while PDT works by producing a large amount of ROS dependent on photosensitizers.^131–133^ Both modalities face challenges such as off-target damage, limited efficacy in hypoxic or deep-seated tumors, and radiation resistance, which can be addressed through hydrogel-based delivery systems.

In radiotherapy, higher doses at the target zone typically yield better therapeutic outcomes, but can also cause radiation tolerance and damage to surrounding normal tissues.^134^ In recent years, studies have shown that combining hydrogels with radiotherapy can reduce the target radiation dose and decrease damage to surrounding normal tissues, improving targeting, efficacy, and simultaneously reducing side effects. For example, agarose hydrogels loaded with ferrous-gallic acid (FeGA) nanoparticles were used to achieve slow release of FeGA at the tumor site.^135^ The photothermal property of FeGA enabled the hydrogel to stably dissolve under laser irradiation. Simultaneously, FeGA reacted with intracellular H_2_O_2_ to generate hydroxyl radicals with mitochondrial toxicity, thereby inactivating tumor cells and reducing radiotherapy resistance. This synergistic effect potentially lowers the required radiation dose and minimizes systemic damage. The hypoxic environment in solid tumors is another major factor affecting radiotherapy efficacy. Yang *et al.* synthesized an injectable thermosensitive composite hydrogel composed of mPEG–PLGA–PFOA modified with perfluorooctanoic acid and perfluorobutyl bromide.^136^ This hydrogel could continuously deliver exogenous oxygen to the tumor site, thus improving the hypoxic microenvironment and downregulating HIF-1α expression. These changes greatly enhanced tumor cell sensitivity to radiation and inhibited tumor growth, ultimately extending mouse survival time. A retrospective clinical study by Miller *et al.* confirmed that hydrogels used as perirectal spacers in prostate cancer radiotherapy could effectively reduce rectal radiation dose and improve patients' gastrointestinal adverse reactions post-radiotherapy.^137^

In PTT, intravenous injection remains the most common administration route for photothermal agents. However, the intravenous administration of photothermal agents still faces challenges that may limit clinical translation and raise safety concerns. For inorganic nanoparticles such as Ti_3_C_2_ MXenes and gold nanostructures,^138^ potential toxicity arise from the inherent toxicity of heavy metal elements, as well as from their long-term biodistribution, bio-persistence, and potential inflammatory responses due to non-biodegradability.^139,140^ Hydrogel-based localized delivery addresses these issues by forming a depot at the tumor site, which minimizes systemic dispersion, reduces off-target accumulation in vital organs, and enhances the local concentration and retention of the agents, thereby improving the therapeutic effect.^141^ Yao *et al.* developed an injectable, biodegradable theranostic system based on Ti_3_C_2_ nanoparticles and a Ti_3_C_2_-gel composite.^142^ In this system, spindle-shaped Ti_3_C_2_ nanoparticles with a size of approximately 50 nm served as photothermal conversion agents. These were simply mixed with the FDA-approved thermosensitive F127 hydrogel to form a multifunctional photothermal gel system. The composite maintained the reversible thermosensitivity of the gel without compromising the photothermal performance of the nanoparticles, generating sustained high temperatures under irradiation to kill tumor cells, while the nanoparticles remained stable for at least two weeks under low-temperature conditions. *In vitro* and *in vivo* experiments demonstrated that this system could effectively raise the temperature under mild laser irradiation (40–50 °C) to ablate 4T1 breast cancer cells. By enabling *in situ* gelation, the platform prolonged its tumor retention and reduced the nanoparticles' toxicity, while also exhibiting excellent photothermal stability and repeatable treatment capability. Furthermore, a novel class of NIR light-responsive, peritumor-injectable hydrogels was developed for excellent PTT efficacy against orthotopic tongue cancer. This system, formed by chitosan and Ag_3_AuS_2_ nanoparticles, exhibited good biocompatibility and superior photothermal effects.^143^ In an orthotopic tongue cancer model, a single PTT treatment efficiently eliminated the tumor. Importantly, this method caused no side effects to surrounding normal tissues and effectively inhibited tumor recurrence.

The localized delivery *via* hydrogel is also highly beneficial for small-molecule photothermal dyes, such as the FDA-approved NIR dye indocyanine green (ICG), which suffers from rapid systemic clearance, poor photostability, and low tumor selectivity when administered intravenously.^144^ One study developed an injectable alginate composite hydrogel co-loaded with ICG-entrapped perfluorocarbon nanoemulsions and CPT-doped chitosan nanoparticles for the treatment of triple-negative breast cancer.^145^ This design enabled stage-wise photochemotherapy: the perfluorocarbon significantly enhanced ICG's thermostability and singlet oxygen production, allowing effective PTT/PDT upon NIR irradiation, followed by sustained release of chemotherapy from the nanoparticles. This sequential and sustained action resulted in potent tumor inhibition without systemic toxicity over 21 days. In another approach, a stimuli-responsive hydrogel based on cartilage ECM and a diselenide-bridged PEG cross-linker was engineered to co-deliver ICG and DOX.^146^ The hydrogel exhibited dual responsiveness to NIR light and reducing conditions, enabling on-demand drug release. This encapsulation strategy prolonged ICG retention and provided controlled, localized hyperthermia and chemotherapeutic action, leading to significant tumor suppression in HT-29 tumor-bearing mice upon irradiation.

**Fig. 6:**
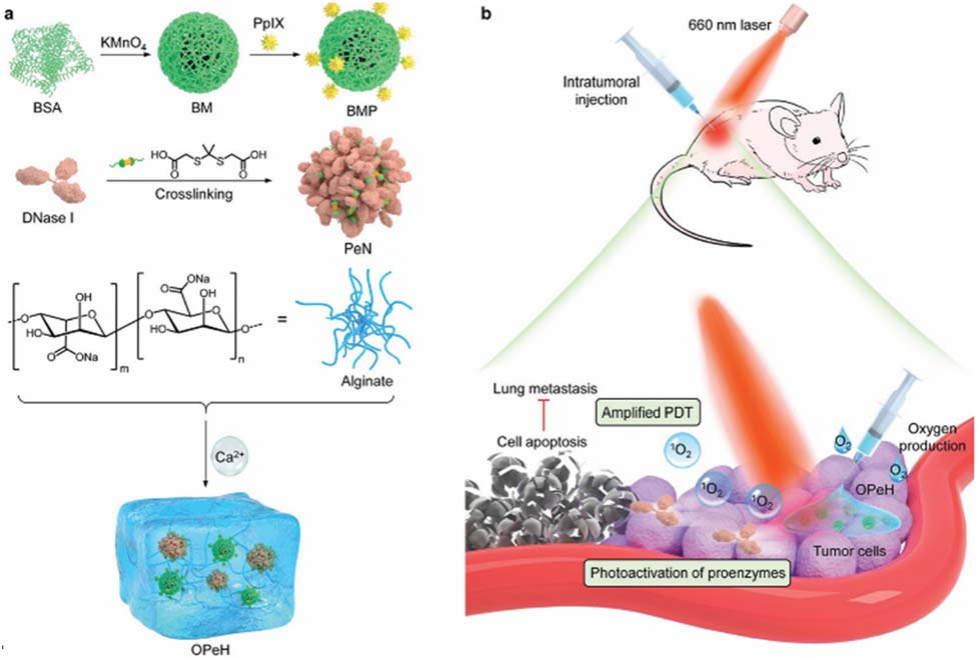
Design of oxygen-producing proenzyme hydrogels (OPeH) for metastasis-inhibiting cancer therapy. (a) Schematic illustration of the preparation of the OPeH. (b) Mechanism of oxygen production and NIR photoactivation of proenzyme nanoparticles for PDT combination cancer therapy, reproduced from ref. 147 with permission from Royal Society of Chemistry (*J. Mater. Chem. B*, 2021), copyright 2021.

In the field of hydrogel-based PDT, a study developed an oxygen-producing proenzyme hydrogel based on photoactivated enzyme catalysis for synergistically enhanced PDT and inhibition of breast cancer metastasis (Fig. 6).^147^ This alginate hydrogel system integrates protoporphyrin IX (PpIX)-modified manganese oxide (MnO_2_) nanoparticles, which function as both a photosensitizer and an oxygen producer, alongside OPe nanoparticles that are responsive to singlet oxygen (^1^O_2_). In the hypoxic and acidic TME, the decomposition of MnO_2_ promoted ^1^O_2_ generation, significantly enhancing the efficiency of PpIX-mediated PDT. From the perspective of material synthesis, hydrogels can be prepared *via* photopolymerization.^148^ For example, a TiO_2_–PEGDA hydrogel system was developed by utilizing the dual functional characteristics of titanium dioxide (TiO_2_).^149^ In this system, TiO_2_ acted both as a photoinitiator for hydrogel formation and generated ROS upon UV exposure to achieve PDT effects. This multifunctional hydrogel possessed antitumor activity and potential as a medical dressing with excellent biocompatibility. Regarding enhancing therapeutic effects, a thermosensitive PF127 hydrogel system loaded with CuS nanoclusters could prolong the photothermal agent's retention time at the tumor site and exert a promising photothermal therapeutic effect in a tumor-bearing mouse model, with low systemic toxicity after peritumoral administration.^150^

Notably, the combination of PDT and PTT can also reduce side effects and exhibit significant synergistic effects.^151^ Yue *et al.* designed a composite hydrogel where carbon dots (CDs) were incorporated into a HA matrix *via* a Schiff base reaction.^152^ This system achieved a singlet oxygen quantum yield of 16% and a photothermal conversion efficiency of 37%. Upon irradiation, the embedded CDs generate heat for PTT and simultaneously produce singlet oxygen for PDT. The HA hydrogel serves as a biocompatible matrix to localize and retain the CDs at the tumor site, thereby ensuring a high local concentration of the CDs, preventing its premature diffusion, and facilitating sustained synergistic action. This excellent optical performance endowed the system with a high antitumor effect in both *in vitro* and *in vivo* studies while maintaining good biosafety. Similarly, Chen *et al.* developed an injectable hydrogel through a Schiff base reaction between amido-modified carbon dots (NCDs) and aldehyde-functionalized cellulose nanocrystals.^153^ In this design, the NCDs serve as both phototherapy agents and crosslinkers. This material exhibited excellent photothermal and photodynamic properties, with a photothermal conversion efficiency of up to 77.6% and maintained a singlet oxygen quantum yield of 0.37 under single irradiation. Specifically, the NCDs generate heat *via* non-radiative transition for PTT while simultaneously sensitizing singlet oxygen (^1^O_2_) production for PDT. *In vivo* animal studies confirmed that this hydrogel possesses good biocompatibility and safety, while efficiently inhibiting tumor growth, demonstrating broad prospects for medical applications.

### 5.5 Gene therapy

Gene therapy involves using gene transfer technology to deliver therapeutic genes into target cells to regulate gene expression, thereby correcting or compensating for diseases caused by gene defects and abnormalities.^154,155^ In recent years, the rapid development of gene therapy technology has revolutionized the field of life sciences. Nevertheless, the advancement of tumor gene therapy is hindered primarily by drug delivery and safety issues.^156,157^ Thus, therapeutic nucleic acids such as antisense oligonucleotides,^158^ siRNAs,^159^ and the CRISPR/Cas system^160^ show great potential in regulating the aberrant gene expression of tumors. However, their clinical translation still faces delivery challenges, including susceptibility to degradation, poor cellular uptake, and off-target effects. To realize this potential, various carriers have been developed. Among these, hydrogels have emerged as a preferred platform due to their excellent modifiability and good biosafety.^161–163^

**Fig. 7:**
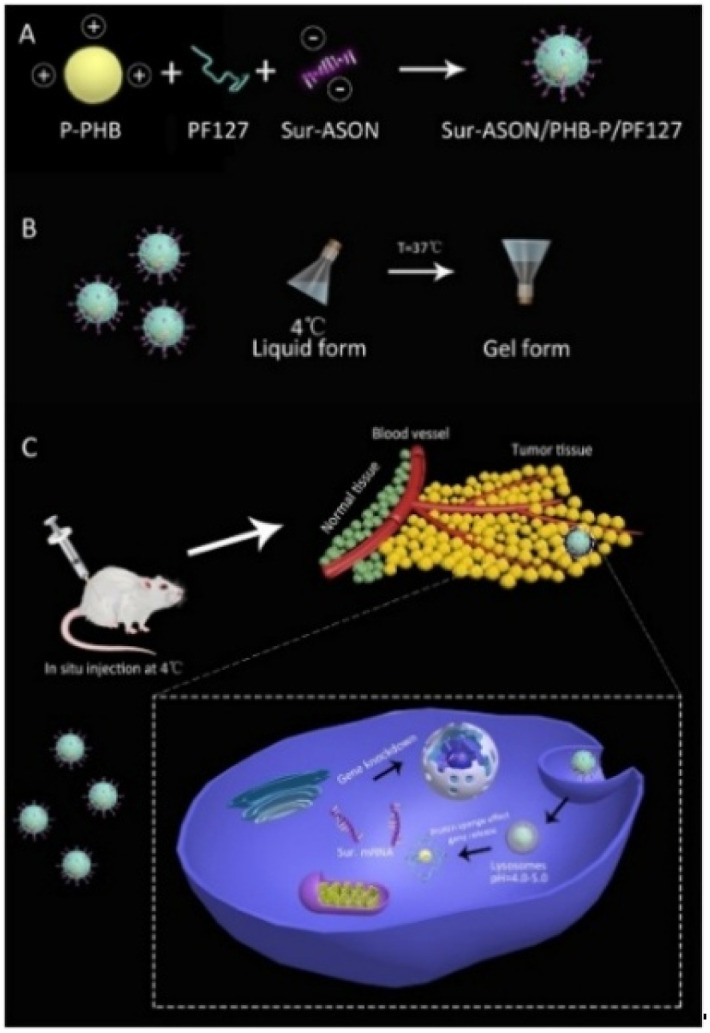
Schematic illustration of the preparation of Sur-ASON/PHB-*b*-PDMAEMA/PF127 hydrogel and *in vivo* therapeutic effect. (A) Formation of Sur-ASON/PHB-P polyplex by electrostatic interaction. (B) Thermo-responsive sol–gel transition of the formulation at 4 °C (liquid) and 37 °C (gel). (C) *In situ* injection into tumor, followed by cellular uptake, endosomal escape *via* proton sponge effect, and intracellular release of Sur-ASON for gene (Sur mRNA) knockdown, reproduced from ref. 166 with permission from Elsevier (*Eur. J. Pharm. Sci.*, 2019), copyright 2019.

Hydrogels offer a promising strategy to address these challenges through multiple mechanisms. First, by encapsulating RNA within their matrix, hydrogels provide a physical barrier that shields it from rapid degradation by nucleases in the extracellular environment, thereby maintaining its bioactivity. Second, the sustained release profile from hydrogels creates a high local concentration gradient of RNA at the target site, which can promote cellular uptake. Furthermore, hydrogels can be co-loaded with or chemically conjugated to cell-penetrating peptides or cationic polymers, which are known to complex with RNA and enhance its endosomal escape and cytosolic delivery.^164,165^ Zhao *et al.* constructed an efficient and easy-to-operate gene delivery system by leveraging the electrostatic complexation between negatively charged survivin antisense oligonucleotides (Sur-ASON) and positively charged PHB-*b*-PDMAEMA (PHB-P) copolymers, combined with a thermosensitive PF127 hydrogel (Fig. 7).^166^ By encapsulating and enabling the sustained release of gene payloads at the tumor site, this system effectively shields them from extracellular degradation. The concurrently released Sur-ASON, complexed with the cationic polymer PHB-P, facilitates cellular uptake and intracellular delivery, thereby significantly enhancing the antitumor effect and inhibiting P-gp-mediated drug efflux. *In vivo* experiments showed that the Sur-ASON/PHB-P/PF127 hydrogel had remarkable tumor-suppressive effects, with enhanced therapeutic efficacy and reduced systemic side effects. This is consistent with the *in vitro* findings where RT-PCR and western blot analysis of MCF-7/PDR cells revealed significantly downregulated expression of both survivin mRNA and protein. Overall, the system achieved controlled gene release for up to 16 days, providing a coordinated strategy to overcome key delivery barriers. Similarly, Gilam *et al.* found that miR-96/miR-182 could downregulate Palladin protein levels and inhibit breast cancer cell migration.^167^ Based on this, they developed a dendrimeric acid–dextran hydrogel loaded with miRNA–gold nanorods system. The implantable hydrogel scaffold acted as both a localizing physical barrier to minimize off-target effects and a sustained-release reservoir for prolonged, site-specific miRNA delivery. The gold nanoparticles were engineered with a targeting peptide (CREKA) to specifically bind to the TME, facilitating receptor-mediated endocytosis for efficient cellular uptake. Once internalized, these nanoparticles likely leveraged endosomal escape mechanisms to release functional miRNAs into the cytoplasm, where they could silence the target gene Palladin. This system efficiently and specifically delivered miR-96/miR-182 to breast tumors, significantly inhibiting primary tumor growth and preventing metastasis.

Furthermore, the combination of gene therapy with other treatment modalities also shows unique advantages. For instance, chitosan@puerarin (CP) hydrogel could be used as a carrier to load gold nanorods (GNRs) and gene-targeted drug DC_AC50, preparing an injectable, stimulus-responsive antibacterial hydrogel (CP@Au@DC_AC50) integrating PTT and gene therapy.^168^ The introduction of GNRs enhanced the mechanical strength of the hydrogel and realized PTT and a thermo-responsive gel–sol transition, which allowed for the on-demand release of DC_AC50 under low-intensity NIR light triggering. The synergistic action of mild PTT and gene-targeted therapy in CP@Au@DC_AC50 efficiently killed tumor cells without damaging normal tissues. This triple-mode therapy (gene-targeted therapy/PTT/antibacterial therapy) triggered by NIR provides a new strategy for constructing multifunctional intraocular tumor treatment platforms.

The CRISPR/Cas system is a revolutionary tool in gene editing and offers precise targeting and high editing efficiency.^169^ This provides new solutions for treating complex tumors involving multiple gene mutations. However, the primary bottleneck for its clinical application lies in the safe and efficient *in vivo* delivery. Recent advances in nanodelivery technology have provided possibilities for this challenge.^170–172^ Chen *et al.* developed a core–shell structured lipid template hydrogel nanoparticles (LHNP) system that successfully achieved efficient co-delivery of Cas9 protein and guide RNA.^173^ By delivering the CRISPR/Cas9 system targeting the model gene Polo-like kinase 1 (PLK1), LHNP effectively inhibited tumor growth and prolonged the survival time of tumor-bearing mice. In another study, a localized co-delivery system using a tuned positively charged hydrogel was devised. This system enabled the simultaneous intratumoral release of a YB-1-knockout CRISPR/Cas9 complex and DOX. The strategy produced a powerful combined effect: YB-1 gene editing sensitized melanoma cells, thereby amplifying the cytotoxicity of the co-delivered DOX. This synergistic action resulted in superior tumor growth inhibition and a reduced side-effect profile, showcasing a promising translational approach.^174^

**Fig. 8:**
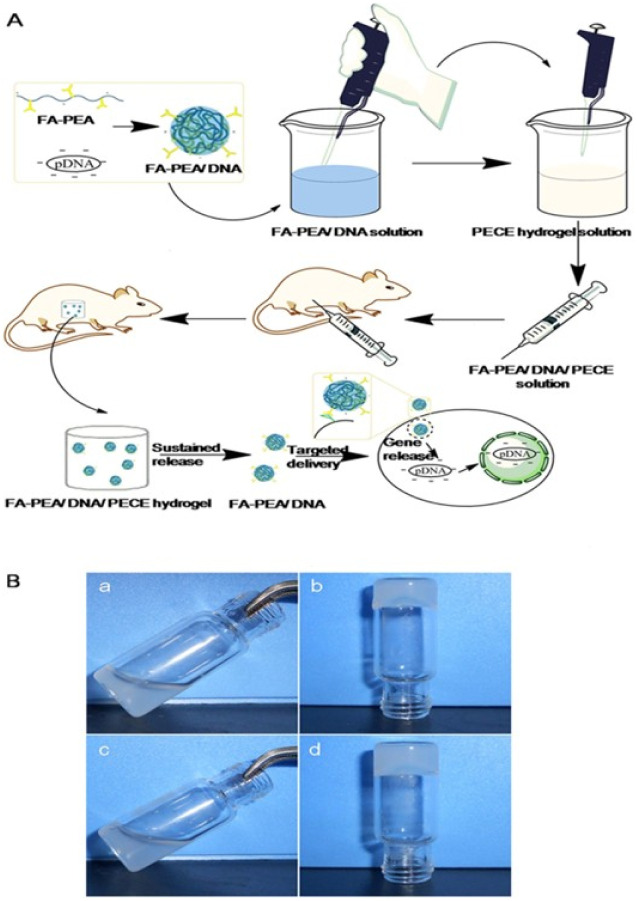
Formation of hydrogel loading gene. Preparation and intracorporal process of hydrogel (A); solgel transition behavior at room temperature and at 37 °C (B), hydrogel (20% weight ratio of PECE) at room temperature (a) and at 37 °C (b), hydrogel (loading FA-PEA/DNA, the weight ratio of PECE was 20%) at room temperature (c) and at 37 °C (d), reproduced from ref. 175 with permission from Springer Nature (*Sci. Rep.*, 2016), copyright 2016.

Targeted delivery is another challenge in gene therapy. Due to the lack of tumor-specific markers and safety concerns, the ability to attain safe and efficient tumor targeting directly impacts the final therapeutic outcome. To tackle this problem, Lin *et al.* reported a DNA-based hydrogel that is capable of encapsulating therapeutic siRNA.^176^ This hydrogel consists of two Y-shaped DNA building units and a fluorescently labeled DNA connector. By utilizing the overexpressed telomerase in tumor cells, which causes hydrogel structure disassembly and fluorescence signal activation, the system allowed for precise telomerase activity detection and *in situ* imaging, while simultaneously releasing siRNA for therapeutic purposes. This DNA nano-hydrogel successfully integrated diagnostic and therapeutic functions, enabling simultaneous telomerase activity detection and antitumor gene therapy. To meet the requirements for tumor-targeted gene delivery, a novel composite was developed by incorporating folate-poly(ester amine) (FA-PEA)/DNA complexes into a thermosensitive PECE hydrogel (Fig. 8).^175^ This system leverages the FA-PEA polymer for targeted delivery and low cytotoxicity, and the hydrogel provides a depot for sustained gene release at the tumor site. This strategy aims to enhance antitumor efficacy by maintaining a high local concentration of the tumor suppressor gene while minimizing systemic side effects. Experiments treated C26 cells with complexes containing the WIF-1 tumor suppressor gene and assessed apoptosis. Results showed that free WIF-1 had no significant effect on C26 cells, whereas cells treated with PEA/WIF-1 and FA-PEA/WIF-1 complexes exhibited apoptosis and necrosis. The apoptosis rate was highest in the PEA/WIF-1 group at 19.4%, compared to 16.5% in the FA-PEA/WIF-1 group and 5.3% in the free WIF-1 group. Furthermore, this system can slowly release the gene into local tissues and selectively deliver it to cancer cells rather than normal cells.

### 5.6 Postoperative adjuvant therapy

Surgical resection remains the first-line treatment for solid tumors. However, the risk of local recurrence and distant metastasis due to residual microtumors and an immunosuppressive postoperative microenvironment poses a major challenge.^177,178^ Systemic adjuvant therapies often suffer from poor biodistribution to the surgical bed and dose-limiting toxicities. Injectable or sprayable *in situ*-forming hydrogels have emerged as an ideal platform to address these limitations. They can fill irregular resection cavities, provide a physical barrier, and function as a localized, sustained-release depot for therapeutic agents directly at the highest-risk site.^179^

Recent advances have demonstrated sophisticated hydrogel designs that go beyond simple drug elution to actively remodel the post-surgical microenvironment. For example, hydrogels capable of delivering chemotherapeutic agents and immunomodulators, either individually or in combination, have shown remarkable efficacy in murine models. They can eradicate residual tumor cells while simultaneously reversing immunosuppression, thereby inhibiting both local recurrence and metastatic spread.^180^ Particularly innovative are multifunctional hydrogels that integrate tumoricidal, antibacterial, and immunoregulatory functions. A prime example is the work by Tao *et al.*, who developed an injectable V-hydrogel assembled from vanadium pentoxide (V_2_O_5_) nanowires and a bactericidal crosslinker (THPS).^181^ This system acts as a localized chemodynamic therapy platform, where the V_2_O_5_ component catalyzes the conversion of endogenous H_2_O_2_ into cytotoxic hydroxyl radicals for direct residual cell clearance, and the THPS provides potent antibacterial protection against surgical site infections. Importantly, the released vanadium ions and the chemodynamic therapy process itself stimulate M1 macrophage polarization and cytotoxic T-cell recruitment, transforming the immunosuppressive post-surgical niche into an immunostimulatory one and establishing systemic immune memory against potential metastases.

Another frontier involves adhesive and hemostatic hydrogels designed for immediate postoperative application. These materials rapidly seal the wound, control bleeding, and locally release agents that target the residual TME. For instance, Cheng *et al.* engineered a sprayable dual-pH-responsive hydrogel for application on liver resection surfaces.^182^ Upon contact, it forms an adhesive gel that achieves rapid hemostasis, reducing the risk of tumor cell dissemination. Beyond this, the hydrogel is co-loaded with a tumor acidity neutralizer and a neutrophil extracellular trap (NET)-degrading enzyme (DNase I). By neutralizing the postoperative acidic milieu and dismantling the pro-metastatic NETs, it effectively reshapes the immunosuppressive wound environment, which in turn significantly enhances the efficacy of co-administered adoptive NK cell therapy, offering a potent synergistic strategy for recurrence prevention.

Furthermore, smart responsive hydrogels tailored to the postoperative environment are being explored. Hydrogels responsive to elevated MMPs or ROS at the wound site can achieve on-demand, feedback-driven drug release, maximizing therapeutic impact while minimizing side effects. Ultimately, the convergence of hydrogel technology with regenerative medicine promises next-generation systems that not only prevent recurrence but also actively promote tissue regeneration and functional recovery at the resection site.^183^

## 6 Localized hydrogel delivery strategies and challenges

The clinical translation of hydrogel therapies depends on effective delivery strategies. While their injectability and biocompatibility are advantageous, the administration route and its associated challenges are crucial for success.

Hydrogel-based therapies are explored across various administration routes, including systemic delivery such as subcutaneous or intravenous injection, and mucosal delivery. However, intratumoral injection is still the most direct and widely investigated strategy for local treatment.^184^ This approach involves the percutaneous delivery of a hydrogel precursor solution directly into the tumor mass or post-resection cavity. Its foremost advantage is the maximization of local drug concentration while minimizing systemic exposure, directly addressing the core goal of reducing off-target toxicity. For injectable systems that gel *in situ* in response to physiological cues, this method allows the hydrogel to conform to irregular tumor geometries, creating a defined drug-eluting depot.^180^ In surgical oncology, intraoperative application presents a synergistic opportunity. Hydrogel precursors can be sprayed, injected, or implanted into the tumor resection bed immediately following excision.^185^ This transforms the surgical cavity into a therapeutic niche designed to eradicate residual microscopic disease, provide hemostasis, and prevent local recurrence.^186^ The rationale here extends beyond simple drug delivery to include physical barrier functions and microenvironment modulation post-surgery.

Despite these advantages, localized hydrogel depots face constraints. The primary limitation of intratumoral or intraoperative delivery is its inherent invasiveness and anatomical dependency. It is unsuitable for disseminated metastatic disease, hematological cancers, or deeply located, inoperable solid tumors where direct access is risky or impossible. Furthermore, the efficacy of a static depot can be compromised by the dynamic and heterogeneous nature of the TME. The variable enzymatic activity, shifting pH gradients, and elevated interstitial fluid pressure within the TME can trigger the premature degradation, burst release, or non-uniform distribution of drugs from a static depot.^187,188^ Another challenge lies in achieving predictable and reliable performance *in vivo*. The gelation kinetics, final mechanical strength, and degradation profile observed *in vitro* may be altered by the complex *in vivo* milieu, affecting drug release kinetics and depot longevity.^189^ Premature dissolution or excessive persistence of the hydrogel carrier can both lead to therapeutic failure.

## 7 Conclusion and outlook

In summary, this review showed the potential of hydrogels as versatile platforms in tumor therapy. By forming a drug-retaining depot at the target site, they address key limitations of conventional treatments such as systemic toxicity and rapid clearance. Incorporating stimuli-responsive features further allows drug release to be activated by specific TME signals, improving both targeting and therapeutic outcomes across various strategies. However, challenges persist in the clinical translation of these sustained-release systems. A major challenge stems from the heterogeneity and dynamic nature of the TME. While stimuli-triggered release mechanisms are effectively demonstrated *in vitro*, their reliability within the dynamically evolving and spatially variable *in vivo* TME remains questionable, directly impacting pharmacokinetics and therapeutic outcomes. Furthermore, achieving optimal synergy in multi-drug regimens requires precise control over the timing and sequence of drug release. This precision goes beyond simple co-encapsulation of drugs and is not routinely achieved by most current delivery systems. Finally, processes such as foreign body reaction, protein adsorption, or unpredictable degradation can alter the long-term fate and biological interactions of the hydrogel matrix itself. This, in turn, may inadvertently change drug release profiles and therapeutic responses.

To overcome these limitations, future research should focus on developing more adaptive and integrated next-generation systems. This includes designing intelligent drug delivery platforms capable of real-time sensing and response to evolving disease biomarkers, as well as engineering hydrogels with sequential or cascading release mechanisms to better replicate complex therapeutic regimens. Advancing *in vivo* pharmacokinetic/pharmacodynamic modeling is also crucial for predicting clinical efficacy. Moreover, materials should be designed for effective biointegration, characterized by minimal inflammatory response and controlled, predictable degradation, thereby ensuring the hydrogel matrix supports rather than hinders the intended therapeutic action. By tackling these challenges in controlled release science, next-generation hydrogel platforms can evolve from passive drug depots into dynamic, bio-integrated therapeutic systems, enabling programmable treatment within the TME.

## Author contributions

Shijia Tian and Shuxiang Yang: conceptualization, investigation, writing—original draft preparation; Yanfei Liu: writing—review and editing, supervision. Both authors approved the final version of the manuscript and agree to be accountable for all aspects of the work.

## Conflicts of interest

The authors declare no conflicts of interest.

## Data Availability

No primary research results, software or code have been included and no new data were generated or analysed as part of this review.
